# The prevalence and correlates of depression and anxiety in a sample of diabetic patients in Sharjah, United Arab Emirates

**DOI:** 10.1186/1471-2296-11-80

**Published:** 2010-10-25

**Authors:** Nabil Sulaiman, Aisha Hamdan, Hani Tamim, Dhafir A Mahmood, Doris Young

**Affiliations:** 1 Department of Family and Community Medicine and Behavioral Sciences, College of Medicine, University of Sharjah, UAE; 2 Department of General Practice, University of Melbourne, Victoria, Australia; 3 College of Arts, Humanities, and Social Sciences, University of Sharjah, UAE; 4 College of Medicine, King Saud bin Abdulaziz University for Health Sciences, King Abdullah International Medical Research Center, Riyadh, Saudi Arabia; 5 Al Qassimi Hospital, Sharjah, UAE

## Abstract

**Background:**

Type 2 diabetes is very prevalent in the Gulf region, particularly in the United Arab Emirates (UAE) which has the second highest prevalence in the world. Factors contributing to this include changes in diet, adoption of sedentary lifestyles, and the consequent increase in rates of obesity. These changes are primarily due to rapid economic development and affluence. The aim of this study was to estimate the prevalence of psychological distress and its correlates in diabetic patients in the United Arab Emirates.

**Methods:**

Patients diagnosed with diabetes attending diabetes mini-clinics in the primary health care centres or hospitals of Sharjah were invited to participate in this cross-sectional study. Patients were interviewed using structured questionnaires to gather data on socio-demographics, lifestyle factors, diabetes complications, and medication usage. The K6 was administered as a screening tool for mental health concerns.

**Results:**

Three hundred and forty-seven participants completed the interview. The majority of participants were females (65.4%) and the mean age was 53.2 (sd = 14.6). Approximately 12.5% of patients obtained a score of 19 or above (cut-off score) on the K6, indicating possible mental health concerns. Twenty-four percent had diabetes complications, mainly in the form of retinopathy, peripheral vascular disease and peripheral neuropathy. A significant relationship was found between scores on the K6, these complications of diabetes and the use of oral hypoglycemic and lipid lowering therapies.

**Conclusions:**

The results of this study demonstrate a strong correlation between mental health status and diabetic complications. In particular, patients who are depressed tended to have poorer self-care, more severe physical symptoms and were less likely to adhere to prescribed care regimens. These findings raise the possibility that improving the mental health as part of a comprehensive management plan for diabetes may improve the overall long term outcomes of these patients.

## Background

Diabetes has been associated with an increased risk of certain psychiatric disorders, particularly depression and anxiety disorders [1]. Patients who have diabetes and a comorbid psychiatric disorder are at increased risk of poorer management and treatment outcomes than those without a psychiatric disorder. Depression has been associated with hyperglycemia [2] and an increased risk for diabetic complications [3] and coronary heart disease [4]. Diabetic patients with depression are also less likely to adhere to medical treatment [5], and more likely to have higher health care use and expenditures than diabetic patients without depression [6].

In a meta-analysis of 39 studies, Anderson et al. found that patients with either type I or type II diabetes had twice the likelihood of experiencing depression (11% and 31% for major depression and elevated depression symptoms respectively) than a matched group of nondiabetic individuals, with the effect generalizing across community and clinical settings [7]. The authors concluded that one in every three individuals with diabetes has depression that impairs their functioning, quality of life and diabetic control.

In a study of diabetes and anxiety in American adults, Barker et al. reported that the overall age-adjusted prevalence of lifetime diagnosis of anxiety was 19.5% for people with diabetes and 10.9% for those without diabetes. After statistical adjustment for educational level, marital status, employment status, current smoking, physical activity and body mass index, people with diabetes had a 20% higher prevalence of lifetime diagnosis of anxiety than those without diabetes [8]. One limitation of this study is that it did not detail specific anxiety disorders.

The World Health Organization (WHO) emphasizes the significance of the relationship between mental and physical health. In their World Health Survey, WHO concluded that depression produces the greatest detriment in health compared with common chronic diseases such as angina, arthritis, asthma, and diabetes. They also reported that the comorbidity of depression with a chronic illness incrementally worsens health outcomes in comparison to depression alone, any chronic disease alone, or any combination of chronic diseases in the absence of depression [9].

A very high prevalence of obesity and Type 2 diabetes has been identified in nationals of the Arabian Gulf countries in comparison to other areas of the world [10 ,11]. According to WHO, Saudi Arabia has the world's third highest rate of obesity (35.6%) followed by the United Arab Emirates (UAE; 33.7%) [12]. Obesity is a major risk factor for the development of diabetes and as such, the UAE has one of the highest prevalence rates of diabetes in the world, second only to Nauru [13]. A recent WHO report on burden of disease estimates that the age-standardized DALYs (Disability-Adjusted Life Years) for diabetes in the UAE is 1,024 per 100,000, one of the highest in the world [14].

In a study on diabetes conducted in the UAE, Saadi et al. [13] randomly sampled households of Emirati citizens in the city of Al-Ain. Of the 2455 adults surveyed, 10.2% reported having been diagnosed with diabetes mellitus. The age-standardized rate for diabetes (diagnosed and undiagnosed) and pre-diabetes among 30-64 year old was estimated to be just over 50% (29% and 24.2%, respectively). A significant proportion of subjects with undiagnosed diabetes and pre-diabetes also suffered from vascular disease.

These high rates are likely related to marked economic development, which has led to a decrease in total mortality and an increase in life expectancy. At the same time, this affluence has lead to the adoption of unhealthy lifestyles resulting in a surge of chronic ill-health, particularly type 2 diabetes and cardiovascular diseases (CVD) [10,15]. These lifestyle changes include decreased levels of activity and increased consumption of calories which contribute to the development of obesity, a major risk factor for diabetes and CVD [13]. The associated physical and mental health complications of these phenomena are expected to have a significant impact in the future [13].

In a study of primary care diabetic patients in Al-Ain, United Arab Emirates, the authors found that 33.8% of diabetic patients had psychiatric co-morbidity [16]. All psychiatric cases were identified as mild or moderate in severity. Depression was the most common disorder (16.9% of diabetic patients) This was followed by mixed anxiety and depressive disorder (9.9%) and generalized anxiety disorder (7.0%) [16].

Despite the high prevalence of diabtes in the UAE, limited attention has been given to the influence of mental health on diabetes-related complications and other diabetes-related variables [16]. It is important to consider this variable due to its potential impact upon disease progression, outcome, and even mortality. The objectives of this study were to estimate the prevalence of psychological distress and to identify its correlates (i.e., demographics/lifestyle factors, diabetes complications, and medication usage) in a cross-sectional study of diabetic patients in Sharjah, United Arab Emirates.

## Methods

### Setting and Participants

The study was a cross-sectional survey conducted in the primary health care centres (PHC) in the Emirate of Sharjah, United Arab Emirates during the period of January 2007 to June 2008. The study was approved by the Institutional Review Board of the Medical and Health Sciences Colleges at the University of Sharjah and was implemented in compliance with the Helsinki Declaration. A research assistant approached patients during the chronic diseases clinic days.She explained the study to potential participants and obtained informed consent. Patients with type 2 diabetes who were 18 years or older were invited to participate. Pregrant women, patients with severe clinical or psychiatric morbidity, patients who were unable to give written consent and those who couldn't communicate adequately in Arabic or English language were excluded. Patients were interviewed using a structured questionnaire on mental health, demographics, life style factors, diabetes complications, and diabetes management. Physical activity questions were based on frequency of a 15-minute vigorous activity and/or 30-minute moderate intensity activity and/or walking during a usual 7-day period [17].

Patients' medical records were reviewed and blood (fasting blood sugar, heamoglobin A1c, cholesterol and triglycerides, urea and creatinin)and urine test results were obtained as well as body weight, height, waist circumference (to the nearest 1 mm), blood pressure and management details.

### Measures

#### Mental Health

The mental health status was assessed using the Arabic version of a widely accepted screening tool, the K6 [18]. This is a six-item self-report scale that assesses the frequency with which an individual experiences symptoms of general psychological distress such as nervousness, tiredness, hopelessness, and restlessness that are associated with serious mental illness. The score for each item ranges from 1 ("none of the time") to 5 ("all of the time"). Summing the unweighted items yields a total score that ranges from 6 to 30. The optimal cutoff point is 6 to 18 versus 19+ [18]. The K6 scale has been validated in the Arabic language [19].

### Statistical analyses

Descriptive analyses were completed by calculating the number and percent of the categorical variables, and the mean and standard deviation (sd) for the continuous variables. Continuous variables were categorized based on clinical reasoning. Bivariate analyses were carried out to assess the associations between two categorical variables by using the Chi-squared test. Moreover, in order to identify the predictors of the mental health, as measured by the K6, we carried out a multivariate logistic regression analyses, where clinically relevant factors were included in the model. In this analysis, odds ratios (OR), and 95% confidence interval (95% CI) were calculated. Association between a categorical variable and a continuous one was carried out by calculating the student's t-test. A p-value less than 0.05 was considered statistically significant. Data management and analyses was carried our using the Statistical Package for Social Sciences (SPSS) version 16.

## Results

Out of 490 patients invited, 347 (71%) participants consented to participate in the study. Non participants were mainly females who refused to participate due to lack of time, confidentiality and literacy. The majority of participants were females (65.4%). The mean age was 53.2 (+/- 14.6) and the mean BMI was 29.8 (+/- 5.9). The large majority were UAE nationals (83.9%), while the remainder originated from various countries including Pakistan (3.5%), Egypt (2.6%) and other Arab countries (10%; i.e., Palestine, Lebanon, Yemen, Iraq, Syria, and Sudan). The majority were married (87.9%). The majority were on oral hypoglycemic medication (80.7%) and 62 (18.8%) patients were on various forms of Insulin (Mixtard, Humilin N, Humilin R). and 24% had established vascular complications affecting mainly the eyes and lower limbs.

### Mental Health Status of Participants

Approximately 12.5% of patients obtained a score of 19 or above (cutoff score) on the K6. While not diagnostic, this score indicates the possible presence of psychopathology in this group of patients, primarily depression or anxiety disorder. Patients in the borderline range may also be at risk of future mental health problems.

### Association of Mental Health with Diabetes-Related Variables

Association between mental health status, as measured by the K6 score, with demographic, lifestyle variables, diabetes-related complications, and medications are presented in Tables 1, 2, and 3. While there were no specific demographic or lifestyle factors significantly related to mental health scores, nationality and marital status bordered significance (p < .05). Nationalities other than Emirati and unmarried patients had higher rates of scores in the 19 and above. It should be noted that a higher level of physical inactivity was found in this sample with 84.1% indicating that they had not walked within the past 7-day. While the relationship was not significant, the trend was toward a higher risk for mental health concerns in those who were inactive.

**Table 1:** Cross-tabulations of Demographics/Lifestyle Variables and Categorization on K-6

	Less than 19		19 and above		
	**n**	**%**	**n**	**%**	**p value**

Sex					
	
Male	99	34.3	14	34.1	
Female	190	65.7	27	65.9	.99

Age					
	
Mean (sd)	53.2	14.6	53.2	15.8	.97

BMI					
	
Mean (sd)	30.1	6.2	28.4	5.1	.13

Nationality					
	
Emirati	246	85.1	30	73.2	
		
Other	43	14.9	11	26.8	.053

Marital Status					
	
Married	254	88.5	34	82.9	.052
		
Not married	33	11.5	7	17.1	

Smoking					
	
Current	10	3.5	1	2.4	
		
Previous	9	3.1	2	4.9	.801
		
Never	268	93.4	38	92.7	

Physical Activity					
	
Yes	46	17.2	3	7.3	
	
No	222	82.8	38	92.7	.108

Family History					
	
Yes	70	28.5	10	27.8	
		
No	167	67.9	22	61.1	.135
		
Not sure	9	3.7	4	11.1	

Age at Diagnosis					
	
Less than 46	53	20.6	10	24.4	
	
46-55	80	31.1	11	26.8	
	
56-65	77	30	8	19.5	.266
	
Above 65	47	18.3	12	29.3	

**Table 2:** Cross-tabulations of Diabetes-Related Complications and Categorization on K-6

	Less than 19		19 and above		
	**n**	**%**	**n**	**%**	**p value**

Eyes^1^					
	
Yes	16	24.2	7	58.3	
		
No	50	75.8	5	41.7	.034*

Feet^2^					
	
Yes	56	53.3	16	88.9	.005*
		
No	16	88.9	2	11.1	

Kidney^3^					
	
Yes	1	1.8	1	16.7	
		
No	54	98.2	5	83.3	.189

Stroke^4^					
Yes	3	5.3	0	0	1.00
		
No	54	94.7	5	100	

Amputation^5^					
	
Yes	2	3.6	1	16.7	
		
No	54	96.4	5	83.3	.267

Heart Problems^6^					
	
Yes	4	6.9	1	16.7	.399
		
No	54	93.1	5	83.3	

Heart Problems-Other					
	
Yes	1	1.9	0	0	
		
No	53	98.1	5	100	1.00

^1^Glaucoma, laser surgery, eye bleeding

^2^Gangrene, loss of sensation/numbness/pain/burning/tingling

^3^Protein or albumin in urine

^4^Loss of sensation one side of body, difficulty speaking/eating, facial drop, paralyzed arm or leg

^5^Loss of toe, foot or leg

^6^Angina, chest pain, heart attack, etc.

**Table 3:** Cross-tabulations of Diabetes-Related Medications and Categorization on K-6

	Less than 19		19 and above		
	n	%	n	%	p value
Oral Hypoglycemics					
	
Yes	205	88.0	32	82.5	
		
No	28	12.0	7	17.5	.032*

Diuretics					
	
Indapamide	29	90.6	4	100	.815
		
Frusemide	2	6.3	0	0	

Betablockers					
	
Atenolol 50	8	53.3	2	66.7	
		
Atenolol 100	6	40	1	33.3	.857

ACE Inhibitors					
	
Yes	61	21.1	7	17.1	.550
		
No	228	78.9	34	82.9	

Receptor blocker					
Losartan	22	78.6	6	85.7	
		
Valsartan	6	21.4	1	14.3	.673

Calcium channel blockers					
	
Amilodipine	16	72.7	1	50	
		
Nifedipine	4	18.2	1	50	.547
	
Verapamil	2	9.1	0	0	

Antihyerlipidemics					
	
Yes	89	30.8	20	48.8	.022*
		
No	200	69.2	21	51.2	

Similarly, the mean K6 score was higher in patients with poorer diabetes control [HbA1c >7 (mean K6 = 11.65) compared with better controlled diabetes HbA1c < 7 (mean K6 = 11.56), but the relation did not reach statistical significance, p = .07]. The mean systolic and diastolic blood pressure followed similar trends, but was not statistically significant. The mean systolic and diastolic blood pressure was 130 versus 135 and 79.2 versus 81.3 in lower and higher K6 scores respectively, but was not statistically significant (p = 0.13 for systolic and p = 0.17 for diastolic).

In relation to diabetes-related complications, two variables were found to be significantly related to K6 scores. These variables were eye-related complications (retinopathy and glaucoma) (p < .034), and vascular/neurological complications in the lower limbs (p < .005). Other complication variables were found to be insignificant due to low numbers. For diabetes-related medications, two categories were found to be significantly associated with K6 scores, oral hypoglycemics and antihyperlipidemics, but not insulin.

### Predictors of Mental health

Table 4 summarizes the results of the multivariate logistic regression analyses. Out of all the variables included in the model, the only significant predictors of K6 being more than 19 was found to be hypertension (OR = 2.53, 95% CI: 1.08-5.92, p-value = 0.03) and complications of the eyes (OR = 3.38, 95% CI: 1.01-11.32, p-value = 0.05). On the other hand, complications in the feet was found to be of border-line significance (OR = 2.24, 95% CI: 0.98-5.11, p-value = 0.06). All the other variables included in the model were not found to be statistically significant.

**Table 4:** Logistic regression analyses for the predictors of *Categorization on K-6*

Variable	OR	95% CI	P-value
**Age**	1.01	0.98-1.04	0.48

**Sex**	1.08	0.50-2.33	0.85

**Nationality**	1.44	0.57-3.62	0.44

**Consanguinity**	1.76	0.62-5.00	0.29

**Marital status**	2.02	0.61-6.75	0.25

**BMI**	0.96	0.89-1.03	0.25

**Systolic Blood Pressure**	1.02	0.99-1.05	0.12

**Diastolic Blood Pressure**	1.03	0.98-1.08	0.23

**Hypertension**	2.53	1.08-5.92	0.03

**Complications: eyes**	3.38	1.01-11.32	0.05

**Complications: feet**	2.24	0.98-5.11	0.06

**Complications: kidney**	3.79	0.19-74.56	0.38

**Complications: amputation**	4.46	0.20-100.72	0.35

**Complications: heart**	2.08	0.16-27.73	0.58

## Discussion

The results indicate that patients with higher scores on K6 having poorer diabetes control than those with lower scores on K6, which is supported by the fact that patients with higher K6 scores (depression and anxiety combined) were more likely to have eye-related complications (retinopathy and glaucoma) and vascular/neurological complications in the lower limbs. These types of complications would correspond to diseases of retinopathy and neuropathy respectively.

The results of this study support research that has been conducted thus far regarding the relationship between mental health and diabetes control and complications. Researchers in the area have concluded that depression, in particular, tends to predict poor diabetes control [20]. In their meta-analysis of 27 studies, de Groot et al. reported a significant correlation between depression and a variety of diabetes complications, including diabetic retinopathy, nepropathy, neuropathy, macrovascular complications and sexual dysfunction [3]. The current research extends these results to an Arab sample in the Arabian Gulf region. These results are significant as minor and major depressive disorders in patients with diabetic foot ulcer have been associated with threefold hazard risk for mortality compared with no depression [24]. Both minor and major depression have been strongly associated with increased mortality overall for diabetic patients [25]. It is important to keep in mind that the direction of the relationship may also be in the opposite direction, in that severe complications may increase the likelihood of depressive or anxious symptoms.

The outcomes of this study highlight the importance of assessing and treating comorbid mental health concerns as part of a comprehensive management plan for diabetes [23]. Treating both co-morbid illnesses simultaneously enhances the likelihood of a successful outcome due to synergistic effects wherein the treatment of one condition affects the course of the other [24]. Due to the cross-sectional nature of the study, it is difficult to determine the exact causal nature of this relationship, but it is likely to be reciprocal. Negative life events, lack of social support, and stress are very likely to aggravate the problems [25].

Patients who are depressed struggle with self-care and adherence to their prescribed care regimens [5,26]. They also often have more severe physical symptoms than patients who are not depressed [3]. This fact was supported by the results of this study, specifically the strong correlation between mental health status and vascular and neurological diabetic complications. As with patients who experience other chronic medical conditions, patients with diabetes often have worse symptoms of depression and respond less well to depression treatment. Depressive symptoms also increase with the severity of the illness [7].

Depressed and anxious patients are more likely to have unhealthy behaviors, such as smoking, overeating, and insufficient physical activities [27]. This would be an additional rationale for targeting depression early in the treatment program. Depressed patients are unlikely to have the energy or motivation to engage in behavior or lifestyle change efforts. Once the depression or anxiety has been addressed, behavior change programs may be initiated. Various cultural factors may influence this variable and may need to be addressed in the management process to enhance compliance [28]. Self-management programs should be initiated in this context and include methods to assist patients with coping with their chronic illness.

While depression and anxiety are common in patients with diabetes, they are often inadequately treated [24]. Effective treatments for depression and anxiety are available, including psychotherapy and pharamacotherapy, so it is important to augment conventional diabetes treatment with these evidence-based therapies that contribute to improved diabetes outcomes [23]. This may enhance glycemic control, reduce the risk of complications and help to preserve the physical health and independence of diabetic patients [23].

One of the challenges in relation to screening, diagnoses and treatment of mental health problems in this region is that mental illness and the utilization of mental health services are highly stigmatized in the culture. This applies to any type of mental health problem, particularly more severe ones, and most types of services (psychiatric, psychological, family and marital therapies) [29]. There is also tendency for somatisation as it is more culturally acceptable to express physical symptoms than psychological [29,30].

The Islamic religion also has a profound impact upon the mental health beliefs and practices of people in the region. For example, people tend to believe that mental illness is associated with supernatural influences, such as *jinn*, the evil eye, or magic, rather than physical or biomedical [29]. Many also believe that a mental illness may be divine punishment as a result of disobedience or sin, or due to weak faith [29]. The prevalent belief in God's will as determining events results in afflicted individuals turning to God in prayer, supplication, religious remedies, or religious leaders, often in disregard to the medical or psychological treatments available [31]. This is an incorrect belief as the various treatments are part of God's decree as well.

There are several limitations of the current study including its cross-sectional nature. This results in difficulty in delineating the causal relationship between the variables. Specifically, diabetes management may lead to the development of depressive symptoms and, in turn, depression may worsen diabetic management and complications. The relationship is very likely to be reciprocal and synergistic. The sample was a non-random, convenient sample and the non response may have introduced bias into the results and limit generalizability. The study also did not include a control group thus limiting its overall power. Patient's literacy level may have affected response due to social desirability or poor understanding of the issues. Finally, it is important to note that the K6 is only a screening tool and is not diagnostic of depression or other psychiatric disorders.

## Conclusion

With these limitations in mind and in conclusion, we have identified a percentage of diabetic patients in Sharjah who are likely to be either depressed or anxious or both. We have also indicated that these mental health problems are more prevalent in patients with diabetes-related retinopathy and neurovascular complications, suggesting a strong correlation between mental health status and diabetic complications. These findings indicate that improving the mental health as part of a comprehensive management plan for diabetes may improve the long term outcomes of these patients. We have highlighted a few strategies that may help physicians deal more efefctively with such patients.

## Competing interests

The authors declare that they have no competing interests.

## Authors' contributions

NS was involved in the conception and design of the project and management of data collection/entry and manuscript writing/revising. AH was involved in the conception and design and manuscript writing/revising. HT conducted data management and statistical analysis and revision of manuscript. DAMA assisted in coordination of data collection and revision of manuscript. DY participated in the questionnaire design as well as writing and revising the manuscript. All authors read and approved the final manuscript.

## Pre-publication history

The pre-publication history for this paper can be accessed here:

http://www.biomedcentral.com/1471-2296/11/80/prepub
